# The *Caenorhabditis elegans* proteome response to two protective *Pseudomonas* symbionts

**DOI:** 10.1128/mbio.03463-23

**Published:** 2024-02-27

**Authors:** Barbara Pees, Lena Peters, Christian Treitz, Inga K. Hamerich, Kohar A. B. Kissoyan, Andreas Tholey, Katja Dierking

**Affiliations:** 1 Department of Evolutionary Ecology and Genetics, Zoological Institute, Christian-Albrecht University, Kiel, Germany; 2 Systematic Proteome Research and Bioanalytics, Institute for Experimental Medicine, Christian-Albrecht University, Kiel, Germany; San Diego St U, San Diego, California, USA

**Keywords:** microbiota, *Caenorhabditis elegans*, *Pseudomonas*, microbiota-mediated protection, proteome

## Abstract

The *Caenorhabditis elegans* natural microbiota isolates *Pseudomonas lurida* MYb11 and *Pseudomonas fluorescens* MYb115 protect the host against pathogens through distinct mechanisms. While *P. lurida* produces an antimicrobial compound and directly inhibits pathogen growth, *P. fluorescens* MYb115 protects the host without affecting pathogen growth. It is unknown how these two protective microbes affect host biological processes. We used a proteomics approach to elucidate the *C. elegans* response to MYb11 and MYb115. We found that both *Pseudomonas* isolates increase vitellogenin protein production in young adults, which confirms previous findings on the effect of microbiota on *C. elegans* reproductive timing. Moreover, the *C. elegans* responses to MYb11 and MYb115 exhibit common signatures with the response to other vitamin B_12_-producing bacteria, emphasizing the importance of vitamin B_12_ in *C. elegans*-microbe metabolic interactions. We further analyzed signatures in the *C. elegans* response specific to MYb11 or MYb115. We provide evidence for distinct modifications in lipid metabolism by both symbiotic microbes. We could identify the activation of host-pathogen defense responses as an MYb11-specific proteome signature and provide evidence that the intermediate filament protein IFB-2 is required for MYb115-mediated protection. These results indicate that MYb11 not only produces an antimicrobial compound but also activates host antimicrobial defenses, which together might increase resistance to infection. In contrast, MYb115 affects host processes such as lipid metabolism and cytoskeleton dynamics, which might increase host tolerance to infection. Overall, this study pinpoints proteins of interest that form the basis for additional exploration into the mechanisms underlying *C. elegans* microbiota-mediated protection from pathogen infection and other microbiota-mediated traits.

**IMPORTANCE:**

Symbiotic bacteria can defend their host against pathogen infection. While some protective symbionts directly interact with pathogenic bacteria, other protective symbionts elicit a response in the host that improves its own pathogen defenses. To better understand how a host responds to protective symbionts, we examined which host proteins are affected by two protective *Pseudomonas* bacteria in the model nematode *Caenorhabditis elegans*. We found that the *C. elegans* response to its protective symbionts is manifold, which was reflected in changes in proteins that are involved in metabolism, the immune system, and cell structure. This study provides a foundation for exploring the contribution of the host response to symbiont-mediated protection from pathogen infection.

## INTRODUCTION

In line with the growing general interest in host-microbiota interactions, *Caenorhabditis elegans* has emerged as a model host to study the effect of different food and microbiota bacteria on host metabolism and physiology. The bacteria used in these studies include bacteria that likely are associated with nematodes in their habitat, such as *Comamonas aquatica*, *Bacillus subtilis*, and different *Escherichia coli* strains [reviewed in reference (1)], and probiotic bacteria of human origin such as *Lactobacillus* and *Bifidobacterium* [reviewed in reference (2)]. The characterization of the *C. elegans* natural microbiome (3, 4) and the creation of the simplified natural nematode microbiota mock community CeMbio (5) initiated a steadily increasing number of recent studies on naturally associated microbes and their interaction with the nematode [reviewed in references (4, 6)]. While we still know relatively little about the function of the *C. elegans* natural microbiota, several studies highlight the important role of the microbiota in supporting the nematode immune response [e.g., references (3, 7–9)].

We previously identified two *Pseudomonas* isolates of the natural *C. elegans* microbiota, which protect the worm from infection with *Bacillus thuringiensis* (*Bt*) through different mechanisms: while *Pseudomonas lurida* MYb11 produces the antimicrobial secondary metabolite massetolide E and directly inhibits pathogen growth, *Pseudomonas fluorescens* MYb115 does not seem to directly inhibit pathogen growth and may thus protect the host by indirect, host-dependent mechanisms (9). The contribution of the host response to MYb11- and MYb115-mediated protection is unclear.

*C. elegans* responses to different food bacteria and natural microbiota isolates have been investigated mainly by transcriptome analyses [e.g., references (10–14)] and only a few proteome analyses (15, 16). Here, we analyzed the direct effects of the protective Pseudomonads MYb11 and MYb115 on the *C. elegans* proteome. To this end, we employed quantitative proteomics and analyzed both commonalities and differences in the *C. elegans* proteomic response to MYb11 and MYb115 and did comparative analyses to previously published microbiota- and pathogen-driven host responses. We validated some of the findings using reporter genes or mutant analyses and thus pinpointed specific proteins that form the groundwork for deeper research into the different molecular mechanisms that underlie *C. elegans*-microbiota interactions, particularly in the context of microbiota-mediated protection against pathogens.

## MATERIALS AND METHODS

### Strains, maintenance, and preparations

Wild-type *C. elegans* N2 and all used *C. elegans* mutants/transgenics, as well as bacteria control *Escherichia coli* OP50, were received from sources indicated in Table S1 and maintained according to standard procedures (17). For each experiment, worms were synchronized by bleaching gravid hermaphrodites with an alkaline hypochlorite solution and incubating the eggs in M9 overnight on a shaker.

Spore solutions of pathogenic *Bacillus thuringiensis* strains MYBt18247 (Bt247) and MYBt18679 (Bt679) were prepared following a previously established protocol (18), and stored at −20°C. Single aliquots were freshly thawed for each inoculation.

*Pseudomonas lurida* MYb11 and *Pseudomonas fluorescens* MYb115 belong to the natural microbiota of *C. elegans* (3) and were stored in glycerol stocks at −80°C. Before each experiment, bacterial isolates were streaked from glycerol stocks onto TSB (tryptic soy broth) agar plates, grown for 2 days at 25°C, and consequently for an overnight in TSB at 28°C in a shaking incubator. One day before adding the worms, bacteria of the overnight cultures were harvested by centrifugation, resuspended in 1× phosphate-buffered saline, pH 7, adjusted to an OD_600_ of 10, and used for inoculation of peptone-free medium (PFM, nematode growth medium without peptone) plates.

### qRT-PCR

Worms were raised on OP50, MYb11, or MYb115 at 20°C until they reached young adulthood, 70 h after synchronized L1s were transferred to the plates. For each replicate, roughly 1,000 worms were washed off the plates with 0.025% Triton X-100 in M9 buffer along with three gravity washing steps. Freezing and RNA isolation were done following the instructions of the NucleoSpin RNA/Protein Kit (Macherey-Nagel, Düren, Germany). A total of 1 µg of the extracted total RNA per sample was reverse transcribed using oligo(dt)18 primers (First Strand cDNA Synthesis Kit; ThermoFisher Scientific, Waltham, USA), and 1 µL of cDNA was used for qPCR with *tbg-1* as housekeeping gene (19). The expression levels of all tested primers were determined using the iQ SYBR Green Supermix (Bio-Rad, Hercules, USA) using the settings as suggested in the manual. Primer sequences are given in Table S2. The 2^-ΔΔCt^ method was used to calculate the relative gene expression (20).

### Survival and lifespan experiments

For survival experiments, synchronized L1 larvae were grown on PFM plates prepared with lawns of OP50, MYb11, or MYb115 at 20°C as described above. PFM infection plates were inoculated with serial dilutions of *Bt* spores mixed with bacterial OP50, MYb11, or MYb115 solutions. As L4s, worms were rinsed off the plates, washed with M9, and pipetted in populations of approximately 30 worms on each *Bt* infection plate. After 24 h-incubation at 20°C, the survival of worms was scored. Worms were considered to be alive when they moved upon gentle prodding with a worm pick. Replicates with less than 15 worms at the time of scoring were excluded.

For lifespan experiments, synchronized L4 larvae were picked onto NGM (nematode growth medium) plates seeded with OP50. Worm survival was determined every day, and the alive adults were transferred to new NGM plates with OP50 until the end of the egg-laying period.

### Worm imaging and quantification

For imaging of *in vivo* gene/protein expression, transgenic worms were treated similarly to survival experiments but without *Bt* infection. Young adults (24 h postL4) were then anesthetized with 10 mM tetramisole, placed onto slides containing a fresh 2% agarose patch, and imaged with a Leica stereomicroscope M205 FA (Wetzlar, Germany). Magnification and exposure time for the fluorophore signal were kept the same in each experiment to ensure comparability; contrast and brightness were adjusted for representative images (grouped worms).

Gene expression of reporter strains was quantified using ImageJ v1.53t (21). Young adults (24 h post-L4) were individually imaged, and the integrated density (IntDen) of each worm was measured. To correct for potential worm size differences, IntDen values were normalized by the total area of each respective individual.

### Proteome analysis

Worms for proteomic analyses were grown on PFM plates prepared with lawns of OP50, MYb11, or MYb115 at 20°C as described above. L4 stage larvae were transferred to freshly inoculated PFM plates to provide sufficient food. Approximately 1,500 worms per replicate were harvested at 12 h post-L4 and washed across a Steriflip 20 µm nylon mesh filter (Merck, Darmstadt, Germany) with M9 buffer. The samples were prepared as four independent biological replicates.

To each sample, 200 µL of protein lysis buffer [100 mM triethylammonium bicarbonate, 2% SDS, 5 M guanidinium chloride, 2 mM dithiothreitol (DTT), and 2× complete protease inhibitor] and approximately 200 µL of acid-washed glass beads were added. The samples were homogenized using a Bioruptor pico for 20 cycles of 30-s sonication and 30-s cooling at 4°C. The protein concentration was determined by BCA assay. The proteins were reduced with 10 mM DTT for 1 h at 60°C and alkylated with 25 mM chloroacetamide at 20°C for 20 min. The samples were centrifuged for 10 min at 10,000 *g,* and aliquots of 100 µg were prepared following the SP3 protocol (22).

A detailed description of the LC-MS analysis is provided in Supplemental Materials and Methods. Briefly, for each of the 12 samples, approximately 1 µg of peptides was analyzed by liquid chromatography-electrospray ionization-mass spectrometry (LC-ESI MS/MS). Proteome digests were separated over a 2 h gradient on a 50 cm C18 nano-uHPLC column, and high-resolution mass spectra were acquired with an Orbitrap Fusion Lumos mass spectrometer. Proteome Discoverer software and the Sequest algorithm were used for peptide identification and label-free quantification. MS data were searched against the reference proteome of *C. elegans* (26,738 entries) combined with the UniParc entries of *P. lurida* (5,392 entries), *P. fluorescens* (5,548 entries), and *E. coli* OP50 (4,227 entries). Statistical evaluation of the quantitative data was performed with the Perseus software (23). LC-MS raw data were deposited to the ProteomeXchange Consortium *via* the PRIDE partner repository (24) with the data set identifier PXD040520.

### Statistical analyses

For the identification of differentially abundant proteins, we performed a one-way ANOVA comparing the three conditions (OP50 vs MYb11 vs MYb115) and corrected for multiple comparisons using a permutation-based FDR analysis. An FDR cutoff of 5% was applied and Tukey’s HSD test was used for *post hoc* analysis. Significant protein groups assigned to each of the pairs of conditions were tested for UniProt keywords by Fisher’s exact test corrected for multiple testing by Benjamini-Hochberg FDR calculation. All significant findings with an FDR below 5% are provided in Table S3.

Heatmaps were created using the Morpheus (https://software.broadinstitute.org/morpheus), and gene ontology (GO) term overrepresentation analyses were done with eVitta v1.3.1 (25). All remaining statistical analyses were carried out with RStudio, R v4.2.1, graphs created with its package ggplot2 v3.3.6 (26) and edited with Inkscape v1.1.2.

## RESULTS

### Common proteomic response to protective *Pseudomonas*

We were interested in identifying the proteomic changes in *C. elegans* exposed to two protective *Pseudomonas* isolates, *P. lurida* MYb11 and *P. fluorescens* MYb115. To this end, worms were grown on MYb11, MYb115, or *E. coli* OP50 and harvested for proteome analysis as young adults. Using LC-MS analysis, we identified 4,314 protein groups in total, which included 259 protein groups annotated to bacterial taxa and 4,055 to *C. elegans* protein groups. For statistical evaluation, the identified *C. elegans* proteins were filtered to 3,456 entries quantified in all four replicates of at least one bacterial treatment. The complete list of proteins is provided in Table S3.

**Fig 1:**
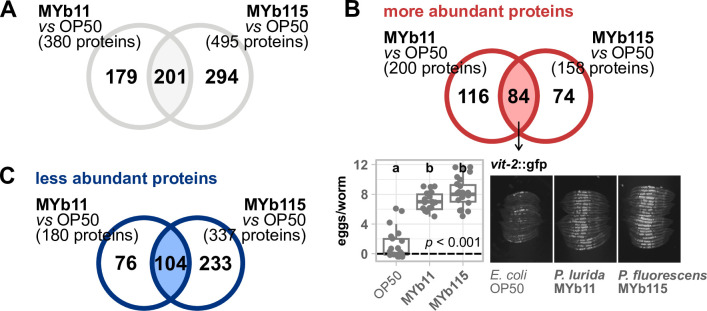
Proteomic response of *C. elegans* toward *Pseudomonas* symbionts. Venn diagrams showing (**A**) all significantly differentially abundant proteins resulting from comparing either MYb11-exposed worms to OP50-exposed worms or MYb115-exposed worms to OP50-exposed worm, (**B**) only the significantly more abundant proteins, or (**C**) the significantly less abundant proteins; ANOVA, *post hoc* Tukey HSD, *P* > 0.05. (**B**) Transgenic *C. elegans* reporter strain *vit-2*::gfp demonstrating *in vivo* abundance of VIT-2. Worms were exposed to either *E. coli* OP50, *P. lurida* MYb11, or *P. fluorescens* MYb115, and gfp signals were imaged in groups of 20 individuals as young adults. Worms were arranged with the heads pointing to the right. The boxplot displays the quantification of VIT-2-expressing eggs/embryos in young adults (24 h post-L4). Each dot represents one worm with *n* = 20, and the dashed line represents the median number of eggs per worm for OP50-exposed worms. The *P*-value indicates the statistical significance among the differently exposed worms according to a Kruskal-Wallis rank sum test (27). The *post hoc* Dunn’s test (28) with Bonferroni correction provides the statistical significances between the differently exposed worms and is denoted with letters (same letters indicate no significant differences). Raw data and corresponding *P*-values are provided in Table S6.

Comparing MYb-treated worms to those grown on OP50, 674 proteins were differentially abundant. Among these proteins, 201 showed a significant difference in both *Pseudomonas* treatments, MYb11 vs OP50 and MYb115 vs OP50 (Fig. 1A; Table S3). When we grouped the shared proteomic response toward *Pseudomonas* into more and less abundant proteins, we obtained 84 highly abundant proteins and 104 less abundant proteins (Fig. 1B and C).

Strikingly, among the more abundant proteins, we found all six vitellogenins described in *C. elegans* (29, 30). Vitellogenins are yolk proteins that are primarily produced in the reproductive phase to supply energy to the embryos (31). Expression of the vitellogenins encoding *vit* genes is known to be greatly upregulated in young adults and downregulated in aging worms (32). We have previously shown that MYb11 and MYb115 accelerate *C. elegans* reproductive maturity without affecting the overall reproductive output (33). Thus, it might be possible that the abundance of vitellogenins in worms treated with either of the Pseudomonads reflects these differences in reproductive maturity. When we compared the abundance of the vitellogenin VIT-2 between young adults on MYb11, MYb115, or OP50 using a *C. elegans vit-2*::gfp reporter strain, we indeed observed an increased number of VIT-2-expressing eggs/embryos and VIT-2 abundance in worms on MYb11 and MYb115 (Fig. 1B).

This observation is reminiscent of data on *Comamonas aquatica* DA1877 and *Enterobacter cloacae* CEent1 that accelerate *C. elegans* development (7, 10).

### Microbiota bacteria elicit a robust proteomic response related to vitamin B_12_-dependent metabolism

**Fig 2:**
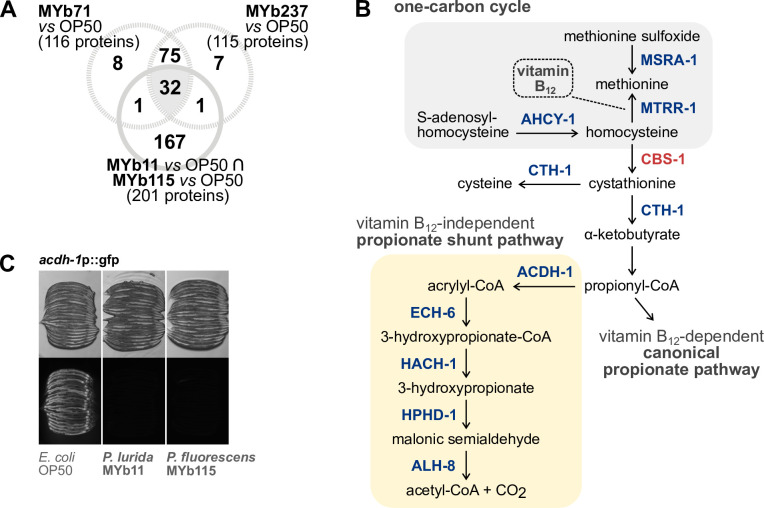
Changes in vitamin B_12_-dependent metabolism are shared proteomic responses to *Pseudomonas* and *Ochrobactrum* symbionts*.* (**A**) Venn diagram showing all significantly differentially abundant proteins resulting from the overlap of the comparison MYb11 vs OP50 and MYb115 vs OP50 compared against differentially abundant proteins on *Ochrobactrum* MYb71 and MYb237. (**B**) Excerpt of the one-carbon cycle (gray background) and the propionate pathways (yellow background). The steps that involve commonly differentially abundant proteins in worms grown on *Pseudomonas* and *Ochrobactrum* symbionts are shown. Protein coloring depicts either less abundant (blue) or more abundant (red) proteins. CoA, coenzyme A. Adapted from references (35, 36). (**C**) Transgenic *C. elegans* reporter strain *acdh-1*p::gfp demonstrating *in vivo* expression of *acdh-1*. Worms were exposed to either *E. coli* OP50, *P. lurida* MYb11, or *P. fluorescens* MYb115, and gfp signals were imaged in groups of 20 individuals as young adults. Worms were arranged with the heads pointing to the right; transmission light images in the upper panel correspond to fluorescence images in the lower panel.

*Pseudomonas* and *Ochrobactrum* represent the most prevalent genera in the natural *C. elegans* microbiota, can colonize the host, and seem to have largely beneficial effects on host life-history traits (3–5, 9, 34). We previously analyzed the effects of *Ochrobactrum vermis* MYb71 and *Ochrobactrum pseudogrignonense* MYb237 on the *C. elegans* proteome (15). Here, we asked whether the *C. elegans* proteome response to the Pseudomonads MYb11 and MYb115 shares common signatures with the response to *O. vermis* MYb71 and *O. pseudogrignonense* MYb237. We extracted significantly differentially abundant proteins in either MYb71 vs *E. coli* OP50 or MYb237 vs *E. coli* OP50 from the published data set and examined the overlap between responses to all four microbiota isolates. We identified 32 proteins, whose abundances were affected by all four microbiota bacteria (Fig. 2A; Table S6). Of the 32 proteins, 31 showed a common increase and decrease in abundances, respectively, relative to the control *E. coli* OP50. One protein, the uncharacterized CHK domain-containing protein F58B4.5, was more abundant in worms fed with either *Ochrobactrum* isolates but less abundant when fed with *Pseudomonas* isolates. It thus represents a promising candidate for understanding contrasting responses to both taxa. We further noticed that 11 proteins out of the 31 proteins representing the common proteome response to *Pseudomonas* and *Ochrobactrum* are members of the interacting methionine/S-adenosylmethionine (met/SAM) cycle, which is part of the one-carbon cycle and the alternative propionate shunt pathway (35, 36) (Fig. 2B). In this signaling network, vitamin B_12_ is a crucial micronutrient that feeds into methionine synthesis and allows the breakdown of propionate (35, 37), thereby promoting *C. elegans* longevity, fertility, development, and mitochondrial health (38, 39).

In the presence of vitamin B_12_, *C. elegans* uses the canonical propionate pathway to degrade propionate into less toxic metabolites and, simultaneously, inactivates the B_12_-independent propionate shunt, i.e., by downregulating the partaking genes (35, 40) (Fig. 2B). Exactly these propionate shunt proteins, ACDH-1, ECH-6, HACH-1, HPHD-1, and ALH-8, were less abundant in the microbiota-treated worms, which is evidence for the provision of vitamin B_12_ by *Pseudomonas* and *Ochrobactrum*. Also, genes encoding the 12 proteins that show different abundances by *Pseudomonas* and *Ochrobactrum* (Table S6) were reported to be differentially regulated by either *C. aquatica* DA1877 or vitamin B_12_ supplementation (35, 36, 41). We confirmed that expression of the acyl-CoA dehydrogenase-encoding gene *acdh-1* is down-regulated by MYb11 and MYb115 by using the dietary sensor *C. elegans* strain *acdh-1*p::gfp, which reacts to vitamin B_12_ presence (35, 42) (Fig. 2C).

### Proteomic responses of *C. elegans* specific to MYb11 and MYb115

**Fig 3:**
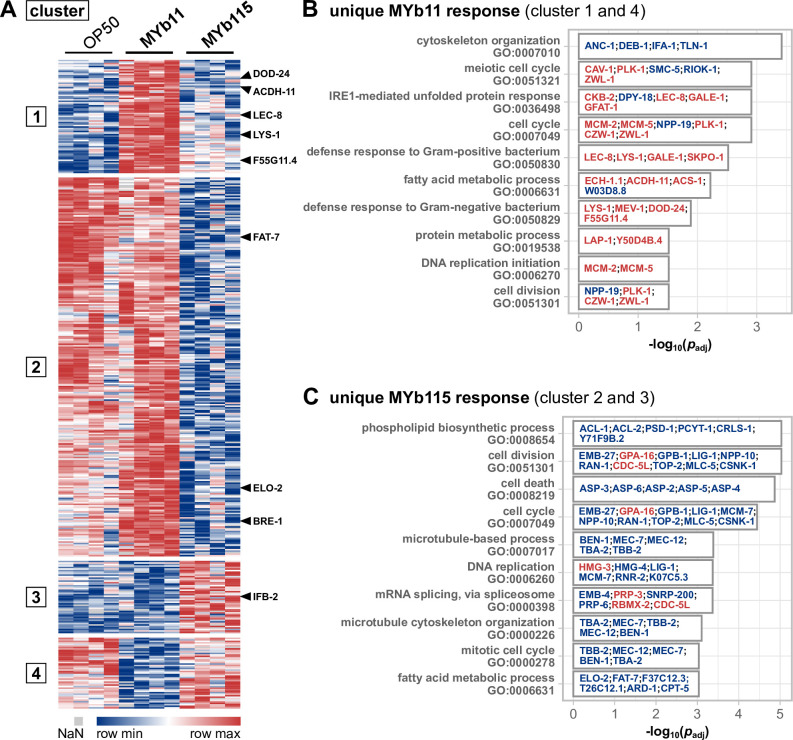
Differences in the proteomic responses of *C. elegans* toward *P. lurida* MYb11 compared to *P. fluorescens* MYb115. (**A**) Heatmap showing the log_2_ label-free intensity values of differentially abundant proteins in the comparison of MYb11-exposed worms against MYb115-exposed worms. The columns denote the bacterial treatment with four replicates each, and each row represents one protein. By including the data on OP50, abundance values were separated into four clusters using the *k*-means clustering approach. The rows of exemplary proteins mentioned in the text are marked on the heatmap’s right. Bar plot of significantly enriched gene ontology terms in either (**B**) clusters 1 and 4 (different abundances of proteins uniquely in MYb11-treated worms) or (**C**) clusters 2 and 3 (different abundances of proteins uniquely in MYb115-treated worms). The proteins that are assigned to the respective GO term are noted on the bars, their coloring indicates higher (red) or lower (blue) abundance. Shown are the 10 GO terms with the highest significance. The complete list of GO terms is given in Tables S4 and S5.

While both Pseudomonads, MYb11 and MYb115, are able to protect *C. elegans* from *Bt* infection, the underlying mechanisms are distinct (9). As a step toward understanding the contribution of the host response to MYb11- and MYb115-mediated protection, we sought to identify the differences in the proteomic responses between worms exposed to MYb11 and MYb115. Both treatments were directly compared, and we found 421 proteins that differed significantly in their abundance between the two conditions (Fig. 3A). Interestingly, 326 proteins were more abundant in worms grown on MYb11 compared to MYb115 and only 95 proteins were more abundant in MYb115-exposed worms compared to MYb11-exposed worms (Fig. 3A). To extract the proteins that were uniquely differently abundant in either MYb11 or MYb115, we included the data on OP50 to generate four clusters using *k*-means clustering: clusters 1 and 4 represent proteins whose abundance only changed in MYb11-exposed worms, i.e., in reference to MYb115 and OP50, while clusters 2 and 3 represent proteins with different abundances specifically in MYb115-exposed worms, i.e., in reference to MYb11 and OP50 (Fig. 3A). Next, we employed eVitta, an online tool developed for the analysis and visualization of transcriptome data (25), to look for enriched GO terms in these clusters.

### MYb11 causes a mild pathogen response in *C. elegans*

**Fig 4:**
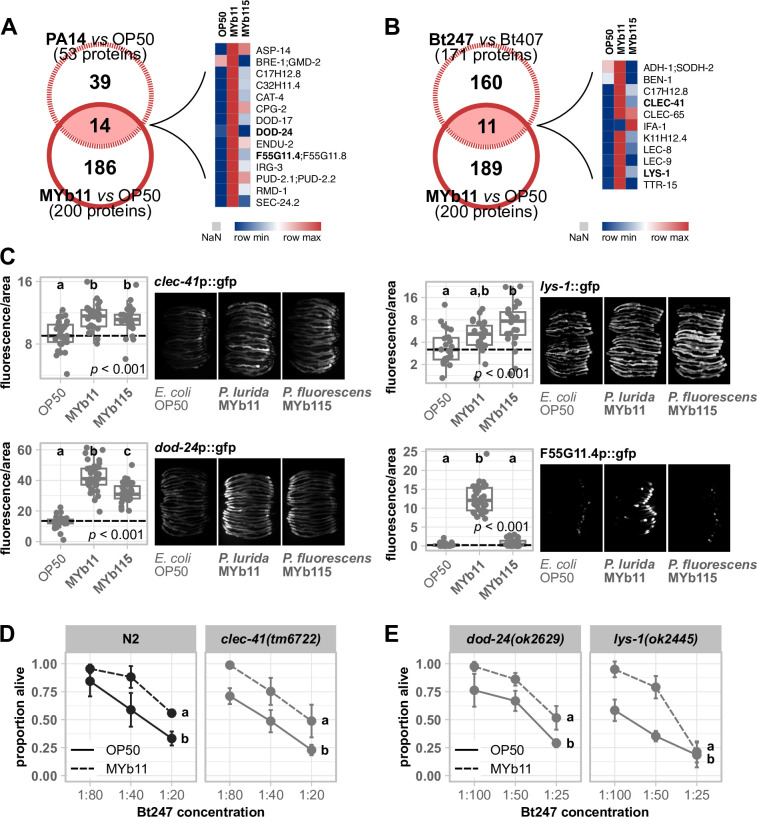
MYb11 activates the expression of *C. elegans* innate immune response genes and proteins. Venn diagrams showing significantly more abundant proteins resulting from the comparison of MYb11 vs OP50 against significantly more abundant proteins of (**A**) *P. aeruginosa* PA14 vs *E. coli* OP50 and (**B**) *B. thuringiensis* Bt247 vs non-pathogenic strain Bt407. The accompanying heatmaps represent the averaged log_2_ label-free intensity values (*n* = 4) of the overlapping significant proteins. Data were taken from references (50, 51). (**C**) Transgenic *C. elegans* reporter strains demonstrating *in vivo* expression of selected promotor sequences tagged with gfp. Transgenic strains were exposed to either *E. coli* OP50, *P. lurida* MYb11, or *P. fluorescens* MYb115, and fluorescent signals were imaged in groups of 20 individuals as young adults. Worms were arranged with the heads pointing to the right. The boxplots display the quantification of the gfp fluorescence in young adults (24 h post-L4) normalized by the worm’s body size (area). Each dot represents one worm with *n* = 29–35, and the dashed line represents the median of the mean gray value for OP50-exposed worms. The *P*-value indicates the statistical significance among the differently exposed worms according to Kruskal-Wallis rank sum test (27). The *post hoc* Dunn’s test (28) with Bonferroni correction provides the statistical significances between the differently exposed worms and is denoted with letters (same letters indicate no significant differences). (**D and E**) Survival of mutants *clec-41(tm6722*), *dod-24(ok262*9), and *lys-1(ok2445*) and wild-type N2 infected with serial dilutions of *B. thuringiensis* Bt247 after 24 hpi (post-infection). Worms were exposed to either OP50 or MYb11, before and during infection. Each dot represents the mean ± standard deviation of four worm populations (*n* = 4). The same letters indicate no significant differences between the dose-response curves according to a generalized linear model (52) and Bonferroni correction. Raw data and corresponding *P*-values are provided in Table S6, and an additional repetition of the experiment (**D**) can be found in Fig. S4.

Proteins affected by both *Pseudomonas* isolates were enriched in GO terms associated with nucleic acids (e.g., DNA replication and mRNA splicing) and also fatty acid-related terms albeit targeting different fat metabolism enzymes (further discussed in next paragraph). On the contrary, defense response proteins were a MYb11-linked feature with defense responses to Gram-positive bacterium (GO:0050830) and Gram-negative bacterium (GO:0050829) among the 10 highest significantly enriched GO terms in the unique MYb11 response (Fig. 3B; Table S4). Interestingly, the seven proteins (LYS-1, LEC-8, GALE-1, SKPO-1, MEV-1, DOD-24, and F55G11.4) associated with the GO defense response terms were all more abundant in MYb11 compared to MYb115 (Fig. 3C; Table S5), indicating that MYb11 induces *C. elegans* pathogen defenses while MYb115 does not. This finding is in line with the previous observation that MYb11 has a pathogenic potential in some contexts, despite its protective effect against *Bt* and *Pseudomonas aeruginosa*, resulting in a shorter lifespan and increased susceptibility to purified *Bt* toxins (33). Interestingly, the lifespan of MYb11-exposed worms on the nutritious medium (NGM) (Fig. S1) is much more decreased than on the minimal medium (PFM), suggesting that the detrimental effect on worms is primarily promoted by proliferating and metabolically active MYb11. Hence, we assessed the general pathogenic potential of MYb11 and MYb115 and tested the activation of the *C. elegans* stress reporters, *hsp-4*::gfp [(43)endoplasmic reticulum stress], *hsp-6*::gfp and *hsp-60*::gfp [mitochondrial stress (44, 45)], *gst-4*p::gfp [oxidative stress (46)], and the immune reporters *irg-1*p::gfp (47) and *clec-60*p::gfp (48) (Fig. S2). Bacteria from the natural *C. elegans* habitat were reported to induce the expression of some of these reporter genes (49). We found that the oxidative stress reporter *gst-4*p::gfp was significantly upregulated only by MYb11 (Fig. S2). MYb11 also slightly induced the expression of the C-type lectin-like gene *clec-60* reporter compared to OP50, but only significantly when compared to MYb115-mediated induction. These results indicate that mainly MYb11 activates the *C. elegans* oxidative stress response and the expression of *clec-60*p::gfp. To explore how far the *C. elegans-*induced proteome response to MYb11 overlaps with the induced proteome response to pathogenic bacteria, we compared our data (Fig. 1B) with the proteomic changes elicited by pathogenic *P. aeruginosa* PA14 (50) and Bt247 (51). The comparison of proteins of higher abundance in MYb11-exposed worms with PA14-responsive proteins yielded an overlap of 14 more abundant proteins (Fig. 4A). Among these 14 proteins were the known pathogen-responsive CUB-like domain proteins C17H12.8, C32H11.4, DOD-17, DOD-24, and F55G11.4, and the infection response gene 3. Similarly, when we compared the response to MYb11 to the proteomic response to *Bt* infection, the abundances of 11 proteins were commonly increased (Fig. 4B). Among these proteins were the CUB-like domain proteins C17H12.8, the lysozyme LYS-1, the galectins LEC-8 and LEC-9, and the C-type lectin-like domain proteins CLEC-41 and CLEC-65. Notably, most of these MYb11- and pathogen-responsive proteins were indeed less responsive to MYb115 (Fig. 4A and B).

Although MYb11 produces the antimicrobial secondary metabolite massetolide E and directly inhibits pathogen growth (9), activation of host-pathogen defense responses, i.e., production of host immune proteins, may contribute to MYb11-mediated protection. To explore this possibility, we focused on F55G11.4, DOD-24, LYS-1, and CLEC-41, whose abundances were strongly increased by MYb11. F55G11.4 was the protein with the highest abundance on MYb11 (Table S3). DOD-24 is commonly used as a marker of the immune response to PA14 and other Gram-negative pathogens (7, 53–55). LYS-1 is required for normal resistance to the Gram-positive *Staphylococcus aureus* (56), and CLEC-41 has demonstrated immune effector function and exhibits antimicrobial activity against Bt247 *in vitro* (57). Mutants of all genes, but F55G11.4, were available at the CGC. First, using qRT-PCR and gfp reporter gene promoters, we confirmed that expression of *dod-24* and F55G11.4 is significantly upregulated by MYb11 in comparison to MYb115 or OP50 also on the transcript level (Fig. 4C; Fig. S3). The expression of the *lys-1* reporter, however, was increased by both MYb11 and MYb115, albeit significantly only by MYb115, and expression of the *clec-41* reporter was significantly induced by both Pseudomonads (Fig. 4C). To determine if these MYb11-induced genes have a function in MYb11-mediated protection against *Bt* infection, we grew the available *dod-24, clec-41, and lys-1* knockout mutants on OP50, MYb11, or MYb115, infected them with Bt247, and scored their survival. MYb11 increased resistance to Bt247 infection also in *dod-24, clec-41,* and *lys-1* mutants (Fig. 4D and E; Fig. S4).

### MYb11 and MYb115 cause diverging responses in *C. elegans* fat metabolism

**Fig 5:**
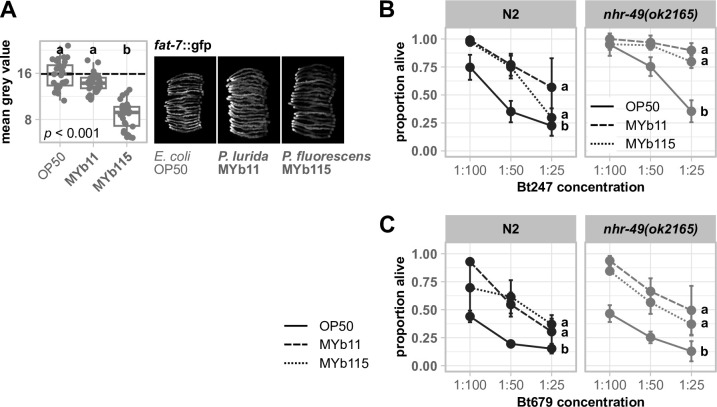
Divergent proteomic changes in fat metabolism occur in MYb11- and MYb115-exposed worms, but common fat metabolism regulator NHR-49 is not involved in the defense against *Bt* infection. (**A**) Transgenic *C. elegans* reporter strain demonstrating *in vivo* abundance of FAT-7. Worms were exposed to either *E. coli* OP50, *P. lurida* MYb11, or *P. fluorescens* MYb115, and gfp signals were imaged in groups of 20 individuals as young adults. Worms were arranged with the heads pointing to the right. The boxplots display the quantification of the gfp fluorescence in young adults (24 h post-L4) normalized by the worm’s body size (area). Each dot represents one worm with *n* = 29–30, and the dashed line represents the median of the mean gray value for OP50-exposed worms. The *P*-value indicates the statistical significance among the differently exposed worms according to a Kruskal-Wallis rank sum test (27). The *post hoc* Dunn’s test (28) with Bonferroni correction provides the statistical significances between the differently exposed worms and is denoted with letters (same letters indicate no significant differences). (**B**) Survival of mutant *nhr-49(ok2165*) and wild-type N2 infected with serial dilutions of (**B**) *B. thuringiensis* Bt247 or (**C**) Bt679 after 24 hpi. Worms were fed with either OP50, MYb11, or MYb115 before and during infection. Each dot represents the mean ± standard deviation of (**B**) four or (**C**) three worm populations (*n* = 3–4). The same letters indicate no significant differences between the dose-response curves according to a generalized linear model (52) and Bonferroni correction. Raw data and corresponding *P*-values are provided in Table S6, and additional repetitions of the same experiments can be found in Fig. S5.

Among the 10 highest significantly enriched GO terms concerning biological processes in the unique MYb11 response as well as in the unique MYb115 response, we found the term fatty acid metabolic process (GO:0006631) (Fig. 3B and C). Moreover, the GO term phospholipid biosynthetic process (GO:0008654) was enriched only in the unique MYb115 response (Fig. 3C). Since the ability to mount an immune response has been repeatedly linked to changes in *C. elegans* fat metabolism [e.g., references (58, 59)], we took a closer look at the underlying proteins. While the predicted fatty acid β-oxidation enzyme ECH-1.1, the acyl-CoA dehydratase ACDH-11, and the acyl-CoA synthetase ACS-1 were of higher abundance in worms on MYb11 (Fig. 3B; Table S3), the fatty acid elongase ELO-2 and the fatty acid desaturase FAT-7 were of lower abundance in worms on MYb115 (Fig. 3C; Table S3). FAT-6 and FAT-7 are members of the long-chain fatty acid synthesis pathway and act redundantly in the synthesis of the monounsaturated fatty acid oleate from stearic acid (60). We validated the effect of the *Pseudomonas* isolates on FAT-7 by assessing the *in vivo* protein abundance of *fat-7*::gfp in worms exposed to *E. coli* OP50, *P. lurida* MYb11, or *P. fluorescens* MYb115. Expression of *fat-7*::gfp was indeed significantly reduced in worms on MYb115 compared to worms on OP50 or MYb11 (Fig. 5A), confirming that MYb11 and MYb115 cause diverging responses in *C. elegans* fat metabolism.

The nuclear hormone receptor NHR-49 is a major regulator of *C. elegans* fat metabolism and activates *fat-7* expression (61). Thus, we evaluated the role of *nhr-49* in the protective effect mediated by either *Pseudomonas* isolate. We tested the survival of the knockout mutant *nhr-49(ok2165*) infected with the *Bt* strain Bt247 or Bt679, in the presence of either OP50, MYb11, or MYb115. Neither MYb11- nor MYb115-mediated protection against *Bt* infection was dependent on *nhr-49* (Fig. 5B and C; Fig. S5).

### Intermediate filament IFB-2 may be involved in MYb115-mediated protection against *B. thuringiensis*

**Fig 6:**
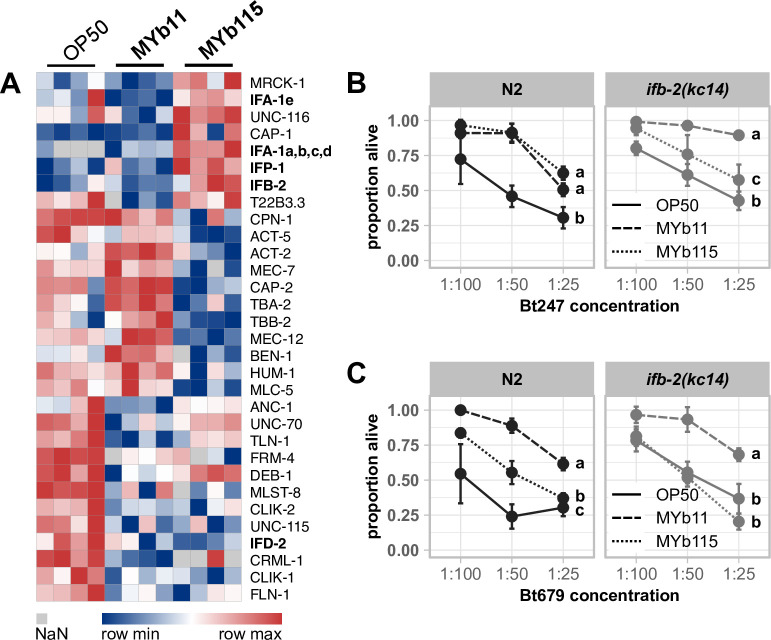
MYb115-mediated protection against *Bt* infection may depend on IFB-2. (**A**) Heatmap showing the log_2_ label-free intensity values of identified proteins related to the GO term cytoskeleton. The columns denote the bacterial treatment with four replicates each, and each row represents one protein. Survival of wild-type N2 and mutant *ifb-2(kc14*) infected with serial dilutions of (**B**) *B. thuringiensis* Bt247 or (**C**) Bt679 after 24 hpi. Worms were exposed to either OP50, MYb11, or MYb115 before and during infection. Each dot represents the mean ± standard deviation of (**B**) four or (**C**) three worm populations (*n* = 3–4). The same letters indicate no significant differences between the dose-response curves according to a generalized linear model (52) and Bonferroni correction. Raw data and corresponding *P*-values are provided in Table S6, and additional repetitions of the same experiments can be found in Fig. S6.

Another intriguing result of our overrepresentation analysis was the presence of cytoskeleton-related terms (e.g., GO:0007010, GO:0007071, and GO:0000226) (Fig. 3B and C). As our previous proteome data set of *C. elegans* infected with *B. thuringiensis* similarly showed enrichment in cytoskeleton-based GO terms (51, 62), we wondered whether systematic reorganization of the cytoskeleton evoked by microbiota members MYb11 and MYb115 might mediate defense against *Bt*. Therefore, we extracted all proteins of our proteome data set with the GO term cytoskeleton (Table S3) and analyzed their abundance pattern (Fig. 6A). Strikingly, four out of five intermediate filaments we identified in the overall analysis, IFB-2, IFP-1, and two IFA-1 isoforms, were more abundant in MYb115-treated worms compared to MYb11- or OP50-fed worms.

The cytoskeleton, consisting of actin-based microfilaments, tubulin-based microtubules, and intermediate filaments (63), canonically stabilizes and maintains the cellular shape [(64); reviewed in reference (65)]. The six *C. elegans* intestinal intermediate filaments, IFB-2, IFC-1, IFC-2, IFD-1, IFD-2, and IFP-1, are all located in the endotube (66), which is positioned at the interface between the intestinal brush border and the cytoplasm (67). To determine the contribution of intermediate filament proteins in the endotube to microbiota-mediated protection against Bt247 and Bt679 infection, we tested the *ifb-2(kc14*) mutant, which completely lacks an endotube (67). We found that the protective effect of MYb115 against *Bt* infection is indeed either partially (Fig. 6B) or completely abolished in the *ifb-2* mutant in four out of five experiments (Fig. 6C; Fig. S6A, B, and D). On the contrary, the MYb11-mediated protective effect seems to be independent of IFB-2 (Fig. 6B and C; Fig. S6C and D).

## DISCUSSION

**Fig 7:**
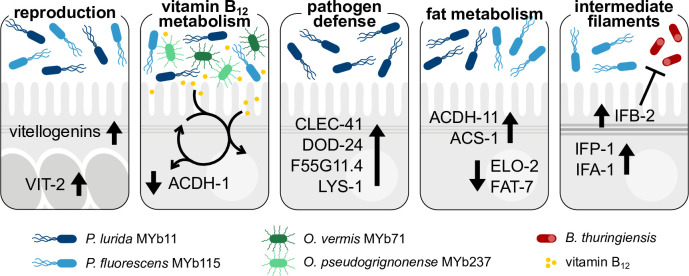
Candidate proteins that are potentially involved in *C. elegans* microbiota-mediated protection. Both *Pseudomonas* isolates, *P. lurida* MYb11 and *P. fluorescens* MYb115, increase *C. elegans* vitellogenin protein production and affect host vitamin B_12_ metabolism. The latter is also affected by other vitamin B_12_-producing microbiota bacteria, such as *O. vermis* MYb71 and *O. pseudogrignonense* MYb237. MYb11 activates host-pathogen defense responses more strongly than MYb115. Moreover, both MYb11 and MYb115 modify host fat metabolism but affect different proteins. MYb115 increases intermediate filament proteins, and MYb115-mediated protection against *Bt* infection was reduced in an *ifb-2* mutant.

This study represents a proteome analysis of the *C. elegans* response to its microbiota members *P. lurida* MYb11 and *P. fluorescens* MYb115, which were previously shown to protect the host against pathogen infection (9). We compared the proteome response elicited by MYb11 and MYb115 with the proteome response to other naturally associated bacteria, to known *C. elegans* pathogens, and directly to each other to reveal common and specific signatures. We thus identified candidate proteins (Fig. 7) that are the basis for further investigation of the mechanisms that mediate pathogen protection.

To reveal common signatures in the *C. elegans* proteome response to naturally associated bacteria, we compared our data with the response to *O. vermis* MYb71 and *O. pseudogrignonense* MYb237, two other members of the *C. elegans* natural microbiota (15). Strikingly, the robust, shared proteomic response of *C. elegans* to *Pseudomonas* and *Ochrobactrum* symbionts seems to be driven by the availability of vitamin B_12_ and subsequent metabolic signaling: 35% of the commonly affected proteins are members of the interacting met/SAM cycle and the alternative propionate shunt pathway (35, 36). Both *Ochrobactrum* isolates, MYb71 and MYb237, and both *Pseudomonas* isolates, MYb11 and MYb115, are predicted vitamin B_12_ producers (34) and our proteomic analysis corroborates this finding. The importance of microbial-derived vitamin B_12_ in regulating the host met/SAM cycle has previously been demonstrated by comparing a *C. aquatica* DA1877 diet, which is naturally rich in vitamin B_12_, to the standard *C. elegans* laboratory food bacterium *E. coli* OP50 (35). Since *E. coli* OP50, which is low in vitamin B_12_, is also commonly used as a control in *C. elegans* microbiota studies, it is important to consider the effect of microbial-derived vitamin B_12_ on *C. elegans* and the resulting, potentially diverse effects on host physiology. For example, vitamin B_12_ was identified as the major metabolite accelerating *C. elegans* development and reproductive timing (35, 39). Moreover, vitamin B_12_ can affect the regulation of host growth, lifespan, chemosensory receptor gene expression, and responses to stress (10, 38, 68, 69). These and other findings stress the importance of microbial-derived vitamin B_12_ in *C. elegans* metabolic processes, which should be considered when studying the effects of the (potentially vitamin B_12_-producing) *C. elegans* microbiota on host physiology.

We are also interested in placing the *C. elegans* proteome response to MYb11 and MYb115 in the context of microbiota-mediated protection against pathogen infection. Both Pseudomonads protect the worm against *Bt* infection, but how far the host response contributes to MYb11- and MYb115-mediated protection remains poorly understood (9, 33). Our proteome analyses revealed several interesting host candidate proteins that may be involved in MYb11- and/or MYb115-mediated protection against *Bt*. First, the abundance of all six vitellogenins described in *C. elegans* (29, 30) was affected by both *Pseudomonas* isolates. In addition to their function in energy supply for the developing embryo, vitellogenins may play a role in pathogen defenses. In the honey bee, vitellogenin drives transgenerational immune priming by binding pathogen-associated molecular patterns of, e.g., *E. coli* and by transporting these signals into developing eggs (70). Also, in *C. elegans*, vitellogenins are involved in defense against *Photorhabdus luminescens* (71). Even more relevant, VIT-2 is required for *Lactobacillus*-mediated protection against methicillin-resistant *S. aureus*, albeit in aging worms (16). Second, as discussed above, both *Pseudomonas* isolates decrease the abundance of proteins of the vitamin B_12_-independent propionate shunt, which indicates that MYb11 and MYb115 provide vitamin B_12_ to the host. Increased vitamin B_12_ availability was shown to improve *C. elegans* mitochondrial health and resistance to infection with *P. aeruginosa* and *Enterococcus faecalis* in a liquid-based killing assay but not to *P. aeruginosa*-mediated slow killing (39). Furthermore, increased vitamin B_12_ availability protects *C. elegans* against exposure to the thiol-reducing agent dithiothreitol (72).

We also identified proteins that were affected by either microbiota isolate. This aspect is of relevance since we know that the protective mechanisms mediated by MYb11 and MYb115 are distinct and that MYb11 and MYb115 have distinct effects on host physiology: MYb11 produces the antimicrobial compound massetolide E and protects *C. elegans* against *Bt* infection directly, while MYb115 does not seem to directly inhibit pathogen growth (9). Also, in contrast to MYb115, which only has neutral or beneficial effects on host physiology, MYb11 reduces worm lifespan (33) and aggravates killing upon exposure to purified *Bt* toxins (33). Thus, MYb11 may have a pathogenic potential in some contexts. In line with this thought, we here found that *P. lurida* MYb11 increases the abundance of known pathogen-responsive proteins, while *P. fluorescens* MYb115 does not. These proteins are commonly referred to as *C. elegans* immune defense proteins, albeit the exact function of the majority of these proteins is unknown. We could confirm MYb11-specific activation of expression of the CUB-like domain-encoding genes *dod-24* and F55G11.4 on the transcript level. Interestingly, F55G11.4p::gfp expression is primarily localized to the first intestinal ring (int1). This observation is reminiscent of the exclusive expression of some *C. elegans* C-type lectin-like genes such as *clec-42* and *clec-43* in int1 (57). The expression of potential immune effectors specifically by int1 might reflect specialization of int1 as the “entry gate” of the intestine, creating a distinct microenvironment that is important for host-microbe interactions.

The increased abundance of immune effector proteins in the presence of MYb11 indicates that MYb11 activates *C. elegans* pathogen defenses. This may reflect its pathogenic potential but may also contribute to its protective effect against *Bt* infection. Demonstrating the involvement of individual immune effectors in microbiota-mediated protection using knockouts of single genes can be challenging due to potential functional redundancy or gene compensation among *C. elegans* immune effectors. Indeed, neither mutant of *dod-24*, *lys-1*, or *clec-41* showed reduced protection by MYb11 upon *Bt* exposure. However, several genes encoding the proteins that we found to be modulated by MYb11 are targets of the *C. elegans* p38 MAPK immune and stress signaling pathway (73–76), and recent work by Griem-Krey *et al.* (77) shows that disruption of p38 MAPK signaling not only abolishes but also completely reverses the protective effect of MYb11 upon infection with Bt679. Thus, we hypothesize that in addition to the production of the antimicrobial compound massetolide E that directly inhibits *Bt* growth (9), MYb11 can protect *C. elegans* from pathogen infection by activating its immune defenses.

While we identified a clear MYb11-specific proteome signature that may contribute to its protective effect, identifying MYb115-specific protein targets with a potential role in protection proved more challenging. We found that both *P. lurida* MYb11 and *P. fluorescens* MYb115 affect *C. elegans* fat metabolism proteins, albeit in different ways. Immune response activation has been repeatedly linked to changes in *C. elegans* fat metabolism. For example, the monounsaturated fatty acid oleate, which is the product of FAT-7 activity, is required for the activation of *C. elegans* pathogen defenses against infection with *E. faecalis*, *Serratia marcescens*, and *P. aeruginosa* (59). Also, the nuclear hormone receptor NHR-49, which is a major regulator of *C. elegans* fat metabolism, mediates *C. elegans* defenses against infection with *E. faecalis* (58), *P. aeruginosa* (78), and *S. aureus* (79). We could show that MYb115 reduces FAT-7 expression. However, our analysis of the *nhr-49(ok2165*) mutant indicates that MYb11- and MYb115-mediated protection against *Bt* infection is independent of *nhr-49*. Thus, the role of *C. elegans* fat metabolism in microbiota-mediated protection against pathogen infection remains to be determined.

The most interesting candidate proteins that we could identify and that may be involved in MYb115-mediated protection are the intermediate filament proteins of the *C. elegans* cytoskeleton. Several intermediate filaments were more abundant in MYb115-treated worms compared to MYb11- or OP50-exposed worms, and the *ifb-2(kc14*) mutant, which lacks an endotube, showed reduced protection by MYb115. In the context of infection, the cytoskeleton functions as a vital barrier against microbial intruders [reviewed in references (80, 81)] but can also be modulated by pathogens to support host colonization [reviewed in references (82, 83)]. We speculate that modulations in cytoskeleton dynamics, i.e., *via* an increase in intermediate filament protein production, by MYb115 might enhance the integrity of the intestinal barrier and thus contribute to defense against pathogens. Indeed, the *Bt* pore-forming toxin Cry5B leads to structural alterations in the *C. elegans* intermediate filament-rich endotube, and the intermediate filament IFB-2 is not only more abundant upon Cry5B exposure but is also required to withstand the detrimental impact of Cry5B (66). Furthermore, the *C. elegans* NCK-1 homolog to human Nck, an activator of actin assembly, was reported to be required for membrane repair after a pore-forming toxin attack (84). Further research is warranted to elucidate the impact of *P. fluorescens* MYb115 on the *C. elegans* intestinal cytoskeleton and its exact role in microbiota-mediated protection against *Bt* pore-forming toxins.

## SUPPLEMENTAL MATERIAL

Tables S1 and S2 mbio.03463-23-s0001.xlsx Transgenic strains and mutants employed in this study; primer sequences for qRT-PCRs.

Supplemental material mbio.03463-23-s0002.docx Supplemental figures and additional experimental details.

Supplemental tables mbio.03463-23-s0003.xlsx Tables S4 to S6.

Table S3 mbio.03463-23-s0004.xlsx Raw data and statistical analyses of proteome data set.

Table S7 mbio.03463-23-s0005.xlsx Raw data and statistical analyses of experiments.

ASM does not own the copyrights to Supplemental Material that may be linked to, or accessed through, an article. The authors have granted ASM a non-exclusive, world-wide license to publish the Supplemental Material files. Please contact the corresponding author directly for reuse.
